# Psychometric Properties of a Standardized Questionnaire of Knowledge, Attitude, and Practice of Iranian Medical Specialists about Viral Hepatitis

**DOI:** 10.5812/hepatmon.7650

**Published:** 2012-12-31

**Authors:** Shahin Ghasemi, Ali Kabir, Mojtaba Ansari Jafari, Mohammad Jalali, Afshin Amini, Amir Hossein Faghihi-Kashani, Seyed Moayed Alavian

**Affiliations:** 1 Rasoul Akram Hospital, Tehran University of Medical Sciences, Tehran, IR Iran; 2 Department of Epidemiology, Faculty of Public Health, Shahid Beheshti University of Medical Sciences, Tehran, IR Iran; 3 Center for Educational Research in Medical Sciences, Tehran University of Medical Sciences, Tehran, IR Iran; 4 Department of Nuclear Medicine, Shahid Beheshti University of Medical Sciences, Tehran, IR Iran; 5 Department of Cardiology, Alborz University of Medical Sciences, Karaj, IR Iran; 6 Imam Hossein Hospital, Faculty of Medicine, Shahid Beheshti University of Medical Sciences, Tehran, IR Iran; 7 Department of Gastroenterology and Hepatology, Tehran University of Medical Sciences, Tehran, IR Iran; 8 Gastroenterology and Hepatology Department, Baqyiatallah Research Center for Gastroenterology and Liver Diseases, Tehran Hepatitis Center, Tehran, IR Iran

**Keywords:** Hepatitis, Questionnaires, Validation Studies, Reliability, Physicians

## Abstract

**Background:**

Good knowledge, attitude and practice (KAP) of the physicians allow them to handle their patients in such a way that they prevent themselves from contracting, and their patients from spreading, the infection. However, the Iranian standardized KAP questionnaire of physicians about viral hepatitis is not available. So, we developed a standard questionnaire.

**Objectives:**

The purpose of this study was to provide a standard questionnaire as a basic tool for assessment of the present situation of the KAP of clinicians. It can also be used for evaluating educational programs and interventions on physicians in addition to any trends in their KAP about viral hepatitis.

**Patients and Methods:**

In order to design and standardize a 29-item self-administered questionnaire, we developed a cross sectional pilot study on 60 Iranian physicians. Ten experts in the field of liver diseases and/or designing the questionnaire answered questions about its validity. Cronbach’s Alpha (on 60 physicians that participated in a congress) and factor analysis (on 370 persons; participants of two viral hepatitis congresses in Tehran and Zanjan and physicians of two university hospitals in Ahvaz) were used in the analysis.

**Results:**

Reliability was 0.7 according to Cronbach’s Alpha score. Face validity was higher than 80%. Content validity of the whole parts of the questionnaire was 96.25% for clarity, 91.56% for relevancy, 96.25% for simplicity and 98.44% for consistency of each question with the questions’ set. Factor analysis showed that 13 components account for 67.4% of the total variance.

**Conclusions:**

This study provided evidence that our questionnaire is a feasible, valid and reliable measure of physicians’ KAP status in Iran. The factor analysis did not reveal a strong cluster structure. This questionnaire should be interpreted as a one-dimensional element by the sum of all items, rather than a multi-dimensional instrument.

## 1. Background

Hepatitis B and C virus (HBV and HCV) are among blood-borne diseases that still pose significant health problems (1, 2) which are easily preventable. Prevalence of HBV and HCV in the Iranian general population is 2.14% (3) and 0.16% (4), respectively. Physicians will visit some cases with HBV and HCV during their careers. Therefore, they should be aware of the principal questions patients are likely to ask them about their diseases: epidemiology of the disease, important risk factors and transmission routes, specifically the ones they encountered the most when in practice. It is important the physicians know a great deal about these diseases and use this knowledge when treating patients infected with HBV and HCV. It is the physician`s attitude and commitment to strong awareness of these diseases that will assist them in teaching their patients and themselves how to prevent the spread of the infection. On the other hand, physicians need to provide their expertise in treating the infected cases. Such a questionnaire could assist health care providers in order to evaluate the adequacy of teaching programs for physicians and evaluate the necessity of retraining. Previous studies revealed that there is an urgent requirement for greater focus on viral hepatitis on the part of Iranian surgeons (5) and dentists (6-8). These previous studies have shown that the knowledge, attitude and practice of Iranian surgeons and dentists are not sufficient and we would require to focus on areas of their weakness and provide education and specific programs to remedy the situation. To evaluate both the areas of weaknesses and assess the need for remedial education, KAP surveys should be performed. Currently, an Iranian standardized questionnaire of knowledge, attitude and practice (KAP) of physicians about viral hepatitis is not presented. Similar questionnaires are also infrequently seen and used in the Middle East region in the field of liver diseases (9-14). Therefore, it is essential that the KAP status of physicians is assessed (and screened) by a standardized questionnaire regarding their KAP with respect to viral hepatitis. Such a questionnaire could be used as the best way to check the awareness, attitude and good practice toward HBV and HCV in physicians. In addition, it can be used by community programmers and policy makers of health-related issues in order to evaluate the requirements for educational programs and potentially necessary interventions.

## 2. Objectives

This article reports the development of an Iranian KAP questionnaire about HBV and HCV and the results of its psychometric testing among physicians with different specialties from different cities in Iran. The marked increase in the survival of patients with viral hepatitis due to medication (15) and liver transplantation (16), together with the effect of the disease on social and family life (17), have led researchers to develop a questionnaire that can be used as a standard tool for further research.

## 3. Patients and Methods

### 3.1. Study Population

We circulated questionnaires among participants in two viral hepatitis congresses: in Tehran (capital city of Iran, a national congress) and in Zanjan (in western Iran, a regional congress) and also among physicians of two university hospitals in Ahvaz (located in southern part of Iran). Participants returned 370 (82%) of the 450 distributed questionnaires. We excluded the results of one questionnaire due to the absence of more than 50% of the data. In this cross sectional study, we considered dentists, general practitioners (GPs), paraclinicians (laboratory specialists, pathologists, anesthesiologists, radiologists, and parasitologists), surgeons (gynecologists, general surgeons, and orthopedic surgeons) and internists (cardiologists, pediatricians, dermatologists, specialists in internal medicine, infectious disease, and emergency medicine) as different groups. This project consisted of different steps for producing and standardizing a questionnaire about KAP of physicians as it relates to hepatitis B and C. Further to designing the questionnaire as a first step, we conducted a cross sectional study of 60 doctors with different specialties, and had them answer the questions in a self-administered manner. Second step was followed by a reliability analysis and factor analysis for reducing the items as much as possible. As a third step, we had 10 experts ensure the validity of the questionnaire.

### 3.2. Designing and Standardizing the Questionnaire

A 29-item self-administered questionnaire assessing risk of transmission; seroconversion rates; the actual prevalence of HBV and HCV in Iran; vaccination against HBV; use of double gloves and protective eyewear; rate of needle stick injury and its reporting; checking the status of viral hepatitis; the use of disposable syringes and how they are discarded; and post-exposure prophylaxis had been designed. The questionnaire used the Likert scale; yes/no and a few open-ended questions, in addition to some demographics (age, gender, specialty, number of hours spent at work per week).

#### 3.2.1. Item Generation

After a thorough search through the available literature, we planned two focus group discussions and one expert panel in order to design flowchart (Appendix 1) and for characterizing the main domains of our KAP survey. Then, we detailed our main domains to some questions. Experts in the field of methodology (epidemiologists; specifically one who was the specialist in designing the questionnaire), a psychiatrist, a specialist in Community Medicine, an infectious diseases expert and gastroenterologists were most important part of our expert panel and focus group discussions.

#### 3.2.2. Item Modification

According to the expert methodologists’ opinions, the structure and the content of some questions had been altered. A pilot study on 60 Iranian physicians with various specialties (participating in the scientific national congresses) was performed to assist in order to modify the structure and content of the primary questionnaire. Answers to each question were also revised according to the different answers to questions in the pilot study.

#### 3.2.3. Item Reduction (Factor Analysis)

Factor analysis provides an enhanced understanding of which variables forms a “relatively coherent subset, independent of others” (18). We performed this analysis on 370 subjects to realize if our most important domains (knowledge, attitude and practice) were categorized by this analysis in the same pattern we first categorized them. We planned to confirm the primary flowchart (Appendix 1) of the questionnaire in this approach.

#### 3.2.4. Item Standardization

##### 3.2.4.1. Reliability

Internal consistency reliability (Cronbach’s Alpha), measuring the extent that the questions in each domain and all three main parts (knowledge, attitude and practice) tap a particular concept (19) was determined according to the pilot study on 60 physicians with various specialties.

##### 3.2.4.2. Face Validity

A separate sample of ten experts in the field of liver diseases and/or designing the questionnaire reviewed the questionnaire and answered the question: “How well do you think the questionnaire measures knowledge, attitude and practice of a physician about hepatitis B and C”? They responded using a 5-point Likert scale from 1 (not at all) to 5 (very well).

##### 3.2.4.3. Content Validity

The content validity of the final questionnaire was determined according to the clarity, relevancy, simplicity, and consistency of each question with the questions set from 10 experts in the field of liver diseases (5 persons) and methodologists (5 persons). They examined the questionnaire for important omissions or inappropriate choice of items. To decrease the prestige bias, demographic variables were inserted at the end of questionnaire. For quality assurance, there was also guidance provided for some questions so the recipient would know how some specific questions should be answered.

### 3.3. Demographic Variables

Age, gender, specialty, place of work, and the quantity of activity in medical practice were among our demographic variables.

### 3.4. Data Analysis

We calculated the internal consistency of the questionnaire using Cronbach’s Alpha coefficient. Factor analysis was done for data reduction and grouping the related variables in conceptually similar and statistically related groups. The extraction method was aprincipal components analysis, the varimax rotation method, and we extracted factors based on an Eigenvalue larger than 1. Kaiser-Meyer-Olkin measure of sampling adequacy and Bartlett’s test of sphericity were used and the cut off point for loading on each factor was 0.3. We used mean ± SD for expressing quantitative variables and correlation test with Pearson coefficient for assessing the relationship between these variables. The analysis was done by SPSS 13 (SPSS Inc. Chicago, Illinois, USA) and Excel software. The authors considered differences and correlations, with *P* < 0.05 being statistically significant. For calculating the Item Content Validity Index (I-CVI), the average of our experts which believed that item is desirable/completely desirable was calculated and expressed as percent. The Scale Content Validity Index (S-CVI) for clarity, relevancy, simplicity, and consistency was calculated as the average of items which our experts believed that were desirable/completely desirable.

### 3.5. Ethics

All subjects signed an informed written consent before participating in the study. The ethics committee of the Baqyiatallah Research Center for Gastroenterology and Liver Disease from Baqyiatallah University of Medical Sciences, Tehran, Iran approved the study proposal.

## 4. Results

The final questionnaire consisted of four parts (Appendix 2): 1. Seven questions about knowledge, 2. Eighteen questions about practice, 3. Four questions about attitudes, and 4. Seven questions about demographic variables.

### 4.1. Demographic Characteristics of Study Participants

Generally, most of the participants were young females. The number of males was 161 (43.6). They had an age average (SD) equal to 40.8 (8.9) and age range between 25 and 79 years, a median duration in medical practice of 12 (range: 0.3 to 50) years, and a median weekly medical practice of 35 (range: 2 to 120) hours. Of the participants, 36.3% were employees of university hospitals and clinics of the Ministry of Health, 39.8% worked in private practice, and 23.9% worked in both private practice and as employees of clinics and university hospitals.

### 4.2. Psychometric Properties

#### 4.2.1. Reliability

Cronbach’s Alpha score, measuring the internal consistency of questions was 0.7. Its value in each domain has been shown in (Table 1).

**Table 1:** Internal Consistency of Different Domains

Domain	Cronbach’s Alpha
**Knowledge**	0.65
**Practice**	0.75
**Attitude**	0.67

#### 4.2.2. Face Validity

All experts rated the question 4 or higher except two persons, producing an overall mean of 4.

#### 4.2.3. Content Validity

The characteristics of the content validity of the whole parts of the questionnaire were clarity: 96.25%, relevancy: 91.56%, simplicity: 96.25% and consistency of each question with the questions’ set: 98.44%. Percent of content validity according to different domains has been shown in (Table 2).

**Table 2:** Percentage of Content Validity According to Different Domains

Domain	Clarity	Relevancy	Simplicity	Consistency
**Knowledge**	98.57	88.57	98.57	95.71
**Practice**	95.24	92.38	96.19	99.05
**Attitude**	97.5	92.5	92.5	100

### 4.3. Factor Analysis

The sample size for the factor analysis was 29 items with 370 subjects. KMO measure of sampling adequacy was 0.557 was considered adequate for the factor analysis, and Bartlett’s test of sphericity also demonstrated a satisfactory suitability of the data to factor analysis (*P* < 0.001) which shows that our variables are related and therefore suitable for structure detection. Extraction communalities are estimates of the variance in each variable accounted for by the components. Our communalities were all above 0.58 and most of them above 0.64. So, all are high which indicates that the extracted components represent the variables well and we do not need to extract another component. A loading cutoff of > 0.30 was adopted, and 13 factors were extracted. Each factor explained 2.98 to 10.39% of the total variance, and 67.4% of the variance was explained by these 13 factors, revealing a weak factor structure. We repeated factor analysis by recoding all variables to three options (even for dichotomized variables). The results did not change significantly.

### 4.4. Findings of the Pilot Study About Main Outcomes

Results are completely explained in a separate paper (20).

### 5. Discussion

Although validation studies could be considered as challenging cases to conduct, their results might be considered worthwhile in various cultures. The first benefit is that they provide standard health measures which allow for health status comparisons between countries. Secondly, they provide validated instruments to monitor population health, to estimate the burden of disease, and to investigate outcomes in clinical practice and to evaluate treatment effects (21). This study provided evidence that our questionnaire is a valid measure of physicians’ KAP status in Iran. Questionnaire was designed to be a self-administered questionnaire, but it could be completed through an in-person interview, computerized administration, or by telephone after small validation studies. Furthermore, the study used a relatively large sample of diverse physicians as defined by different age groups, gender, specialties, expertise, cities of practice, duration of practice and location of practice (clinic and/or hospital, and private and/or governmental). Therefore, the standardized questionnaire acquired from this study could reliably be used for all Iranian physicians. In general, all psychometric tests of the questionnaire showed satisfactory results. Reliability of the questionnaire as measured by the Cronbach’s Alpha coefficient for all three scales and all parts at once exceeded the recommended level. With I-CVI and S-CVI, all percentages were higher than 80% which are considered as the minimum acceptable for a new tool (22-25). Therefore nearly all of them were acceptable and such a questionnaire can be used in similar situations (Iranian physicians about viral hepatitis). It may also be used in other Iranian cases which do not have significant differences affectingthe validity of this questionnaire. The factor analysis did not reveal a strong cluster structure, suggesting that the questionnaire should be interpreted as a one-dimensional element by the sum of all items, rather than as a multi-dimensional instrument. In general, the questionnaire should be interpreted with the sum of all items rather than as isolated items or clusters of questions. Factor analysis confirmed that there are not limited numbers of questions that can explain most of variance of the questionnaire. Thirteen factors explained 67% of the variance and shows that we have truly split the different parts of KAP into subtitles with the least overlap. Factors were related to the same parts of KAP. The significance level of Bartlett’s test was less than 0.05 in our study which indicates that a factor analysis may be useful with our data. Few questionnaires of this type have published data on their validity and reliability and we did not find a similar one to which we could compare our results. So, we compared our findings with standard indices like 0.7 for Cronbach’s Alpha and 80% for CVI. Obviously, we could have a translation of an existing questionnaire. However, since translating a questionnaire is strenuous and a translation might not be suitable for all subgroups in view of medical terminology and practices as well as general knowledge about hepatitis differ among populations. One limitation of this study is that although our research began with careful operationalization of the domain of viral hepatitis-related knowledge, attitude and practice of physicians with a rational item selection phase, the resulting measure cannot be considered a comprehensive measure of viral hepatitis-related KAP. Although the questionnaire emphasizes some more important parts, it does not comprehensively assess all risk factors in details. Moreover, the measure does not attempt to assess knowledge regarding the natural history, clinical course, or treatment of viral hepatitis. Thus, in applications or settings where a more comprehensive assessment is needed, we recommend expenditure of a longer and in more detailed questionnaire. However, it could be mentioned that longer questionnaires have a lower validity and reliability because of a diminution in the attention of the responders. Although our study did not provide evidence for test-retest reliability, responsiveness to change or other psychometric tests, the findings showed that this questionnaire is a reliable appraise for measuring physicians’ KAP about viral hepatitis. Future studies could focus on other psychometric properties of the questionnaire and also on different applications of the questionnaire. In conclusion, although the present study does not provide evidence on test-retest reliability or on responsiveness, the findings however provide further evidence that this questionnaire about viral diseases is feasible in Iran and could be used as a reliable and valid instrument for measuring KAP of physicians. It could be used in several academic research projects and further results from its validity are anticipated.

## Appendices

### Appendix 2 Questionnaire of Knowledge, Attitude, and Practice of Iranian Medical Specialists About Viral Hepatitis

In the name of God

Dear Colleague

This questionnaire with 36 queries has been designed to measure knowledge, attitude and practice of Iranian physicians about protection of their patients and themselves from hepatitis B and C. The results are useful in designing knowledge and performance improvement programs, and protecting patients' and physicians' safety and health.

There is no need to mention your name. Please fill blank places carefully and tag in your desired squares. Your higher precision in answering will help us to achievement of our goals.

**Table:**

**Are you active in medical practice at present**		
Yes		
No		
**Years in practice?**		......years
**Average number of hours in medical practice per week?**		......hours
**Do you wear double glove, when operating?**		
Usually		
	Rarely	
Never		
	Do not surgery	
**Have you ever tried a period of double gloving?**		
Yes		
No		
at all	Do not surgery	
**Do you use mask/glasses, when operating?**		
Always		
Occasionally		
Never		
**What percentage of your patients do you believe are positive for**		
Hepatitis B		.....percent
Hepatitis C		.....percent
**What percentage of your patients do you order blood tests to screen for**		
Hepatitis B		.....percent
Hepatitis C		.....percent
**How many dosage have you ever been vaccinated against hepatitis B?**		
Zero		
Two		
Three		
**Date of last dosage: (mm/dd/year)**		.../.../...
**Where the interval of vaccination according to the protocol (0, 1 and 6 months)?**		
Yes		
No		
**If you have vaccinated, have you checked your antibody level after vaccination?**		
Yes		
**If you have checked the titer, was it positive:**		
Yes		
No		
**If you did not complete the vaccination course (3 dosages), what are the causes?[you can select more than one option]**		
I am completing my course of vaccination		
I gave up vaccination		
I suppose I have complete immunity (without checking antibody)		
**Due to the probability of complications**		
**Age higher than 45 years old is the contraindication of vaccination**		
**Others (cost issues, time limitation and etc.)**		
**Do you use disposable syringe?**		
Yes		
No		
**Do you recap the needle before discarding?**		
Rarely		
Never		
**How discard needles after usage?**		
In carton box		
In plastic box		
In garbage bags		
Other: (Please specify)		
**Do you have any microabrasions or open sores/cracks on your hands or nailbed region?**		
Always		
Sometimes		
Occasionally		
Rarely		
Never		
**How often do you report an actual needle stick injury?**		
Always		
Sometimes		
Occasionally		
Never		
**How many needle stick injuries have you had in the last 3 years?**		.....times
**Have you ever been stuck by a needle while treating a patient positive for**		
**Hepatitis B**		
Yes		(... # of times)
No		
**Hepatitis C**		
Yes		(... # of times)
No		
**Have you ever had eye/mucosa contact with secretions while treating a patient positive for**		
**Hepatitis B**		
Yes		(... # of times)
No		
**Hepatitis C**		
Yes	(... # of times)
No		
**Secondary to a needle stick injury, have you ever been treated with**		
**Vaccine**		
Yes		(... # of courses)
No		
**Immunoglobulin**		
Yes		(... # of courses)
No		
**No need according to the specialist comment**		
**Not binding to the prevention guidelines will increase the probability of: [you can select more than one option**		
**Patient to patient infection transmission**		
**Patient to physician/dentist infection transmission**		
**Physician/dentist to patient infection transmission**		
**Transmission of hepatitis B and C is possible from saliva**		
Yes		
No		
**Transmission of hepatitis B and C is possible from dentistry**		
Yes		
No		
**The probability of hepatitis B infection is higher than HIV after Needle stick Injury**		
Yes		
No		
**The prevalence of hepatitis B is higher than 1% in Iranian population**		
Yes		
No		
**The prevalence of hepatitis C is lower than 1% in Iranian population**		
Yes		
No		
**Using gloves is effective for preventing hepatitis B infection**		
Yes		
No		
**What is the serum conversion rate secondary to a needle stick injury from a patient positive for**		
**Hepatitis B?**		..... percent
**Hepatitis C?**		.....percent
**Do you concern about infection with: (Score from 100 as highest concern)**		
**Hepatitis B**		
**Hepatitis C**		
**Have you checked for these infections during three last years?**		
**HBV**		
Yes		
No		
**HCV**		
Yes		
No		
**Specialty/Sub-specialty (fellowship)**		
**Place of working**		
Governmental		
Private		
Both		
**Age**		.....years
**Gender**		
Male		
Female		
